# Effect of Collagen Coating and Fiber Profile on Tenocyte Growth on Braided Poly-ε-Caprolactone Scaffolds for Tendon and Ligament Regeneration

**DOI:** 10.3390/ijms26041735

**Published:** 2025-02-18

**Authors:** Caroline Emonts, Benedict Bauer, Charlotte Büchter, Thomas Pufe, Thomas Gries, Mersedeh Tohidnezhad

**Affiliations:** 1 Institut für Textiltechnik, RWTH Aachen University, 52074 Aachen, Germany; benedict.bauer@ita.rwth-aachen.de (B.B.);; 2 Department of Anatomy and Cell Biology, RWTH Aachen University, 52074 Aachen, Germany

**Keywords:** tendon, ligament, tissue engineering, scaffold, PCL, tenocyte, collagen, fiber profile, cross-section, braiding

## Abstract

Using scaffolds is a promising alternative to current methods of treatment for ruptures of tendons and ligaments. However, scaffolds are subject to a wide range of challenges, including mechanical, degradation, process-related and biological requirements. Poly-ε-caprolactone (PCL) fibers have already shown potential for tendon tissue engineering (TTE) because of their degradation kinetics and excellent mechanical properties. The objective of this study was to enhance the PCL scaffold for TTE, specifically in regard to the filament morphology and collagen coating. PCL fibers were melt-spun as monofilaments with circular and snowflake-shaped cross-sections. Different scaffold densities were achieved by applying three different braiding angles in the braiding process. Morphological characterization was conducted including porosity and pore size distribution using µ-CT. The scaffolds were collagenized and cellularized with primary tenocytes for 7 days. Immunofluorescence staining showed a certain alignment of cell growing direction with fiber direction. In cell viability and cell proliferation assays, significant improvements in cell response were observed for the snowflake fiber and collagen coating groups, especially when combined. The data suggest that the utilization of non-circular fibers may facilitate enhanced cell guidance and surface area, while the application of a collagen coating could optimize the cellular environment for adhesion and proliferation.

## 1. Introduction

Tendon and ligaments are hypocellular and hypovascular dense connective tissues. Therefore, these tissues often require assistance for restoration after a complete tear [1,2,3,4]. The current gold standard is the use of autografts. This method is accompanied by limited graft availability, donor site morbidity, and longer surgery times. Available synthetic grafts are rarely used as complications occur such as poor host tissue integration, a lack of mechanical stability, or long-term wear resistance [5].

For these reasons, the field of tendon and ligament tissue engineering is a rising research field. Tendon and ligament tissue engineering aims at the development of suitable scaffolds that could support the regeneration of the native tissue after damage. There are numerous requirements for the scaffold from a mechanical and biological point of view as well as with regard to the degradation profile. The simultaneous achievement of these requirements remains a major challenge.

Tendon and ligaments have a hierarchical structure starting with collagen fibrils forming fascicles and bundles [6]. To mimic this structure, fibrous and textile scaffolds are especially suitable. Textile structures offer good reproducibility, scalability, and 3D structures [7]. The structure can be designed so that the fibers are arranged in an optimized way according to the occurring physiological loads. Tendons and ligaments are mostly tensile-loaded structures. Therefore, braids offer a suitable scaffold structure, which is load-efficient and capable of high strength because all fibers are mostly oriented in the direction of the tensile load. Depending on the application the mechanical properties can be adjusted in a wide range via the braiding parameters, i.e., number of filaments, braiding angle, braiding pattern, and braiding geometry [8,9]. The aforementioned parameters can also be used to modify the morphological parameters including pore size and porosity as well as diameter and width.

The biomimetic design of braided scaffolds for ligament tissue engineering was shown by Cooper et al. using poly(lactide-co-glycolide) (PLGA) fibers and 3D-braiding methods. The variability of porosity and pore size was demonstrated by adapting to bone and ligament tissue [10]. The biological suitability of these scaffolds was shown by Lu et al. [11]. Also, circular braiding was used in several studies for the scaffold production of degradable anterior cruciate ligament (ACL) scaffolds. Laurent et al. investigated poly(lactide-co-ε-caprolactone)-based braided ACL scaffolds by simulation and experimental methods [12]. The multilayer-braided scaffold was also functionalized by poly-L-lysine/hyaluronic acid to enhance the cell interaction of mesenchymal stem cells [13]. Freeman et al. manufactured scaffolds for ligament replacement by braiding, twisting, and braid-twisting poly(L-lactic acid) (PLLA) fibers. The mechanical properties as well as the biological analysis showed potential in these biomimetic scaffold structures [14]. The possibilities of braiding technology to produce tissue-engineered ligaments is demonstrated by these studies.

A variety of synthetic polymers have been used to prepare fibrous scaffolds in tissue engineering of tendons and ligaments. To enhance the attraction of the mostly hydrophobic surface of the synthetic polymers for cell growth, these polymers are often combined with natural materials such as collagen. Collagen is one of the main components of native tendons and ligaments. Exemplary, the ECM of the ACL is composed of more than 85% of collagen type I and III (related to dry tissue) [15].

In multiple studies, collagen has successfully been used to improve cell adherence on fibrous scaffolds made of synthetic polymers. Gögele et al. studied P(LA-CL)/PLA scaffolds manufactured by embroidery and functionalized by gas-phase fluorination and a cross-linked collagen foam. The functionalization allowed a higher cell colonization and cell migration into the three-dimensional structure [16]. Ide et al. evaluated the cell behavior on PLLA–collagen hybrid scaffolds for ACL replacement. A three-dimensional braid of PLLA fibers was infiltrated with collagen. The hybridization facilitated the cell seeding with human ACL-derived cells. Furthermore, cell migration and neo-angiogenesis were promoted in the in vivo rat model [17]. Collagen has also been investigated in combination with natural materials such as silk. Sharifi-Aghdam et al. combined knitted silk scaffolds with blended collagen and electrospun polyurethane fibers. Therefore, cellular attachment and proliferation were improved [18]. Also, Zheng et al. used a knitted silk scaffold for rotator cuff repair and molded it in collagen hydrogel to create a macroporous 3D-aligned scaffold. The in vivo study showed a more efficient cell recruitment of this scaffold compared with a sponge collagen/silk scaffold [19].

Besides functionalization of the surface, the topography is also crucial for cell adhesion and can trigger the differentiation of stem cells in combination with biochemical stimuli [20]. The alignment of PCL fibers enhanced the expression of tendon-specific genes [4,21,22,23]. Another option for topographical changes in the scaffold surface are fibers with non-circular cross-sections. This approach has rarely been studied to date in the context of tendon and ligament tissue engineering, not to mention applied on PCL fibers. This option was investigated by Ramakrishna et al. using investigated PLA multifilaments with four deep grooves (4DG) that were combined with round PLA multifilaments to form a scaffold for the bone–tendon junction. Compared to a scaffold consisting entirely of round PLA multifilaments, the integration of the 4DG-fibers resulted in a marginal improvement of murine TGF-β-type-2 receptor (Tgfbr2)-expressing progenitor cell proliferation. Furthermore, attachment and alignment of the cells within the grooves of the 4DG fibers were observed [24]. Lu et al. observed that the majority of cell nuclei (>85%) aligned in parallel with the channels in their fibers from polylactic acid (PLA) and polyethylene terephthalate (PET) [25]. Park et al. investigated the effect of different morphologies (circular, triangle, and cruciform) of extruded PCL strands on cell proliferation. After 7 days of cultivation, the triangular and cruciform samples showed 50% and 112% increased cell proliferation (MG-63) compared to the circular reference, respectively [26]. Summarizing, cell proliferation, attachment, and/or alignment can be affected using topographical cues. However, to date only a few studies are designed to take advantage of these approaches.

In previous studies, we showed the development of the melt spinning process of the PCL fibers with a high draw ratio [27]. Furthermore, we demonstrated the suitability of these PCL fibers for scaffolds for ACL replacement using circular and 3D hexagonal braiding [27,28,29,30]. Since the surface of the scaffold is the first contact for the cells, bioresponsiveness of the surface through suitable topography and functionalization may contribute to improved interaction between cells and material. The focus of the present study was to investigate the influence of the surface topology of PCL melt-spun fibers and collagen functionalization of braided scaffolds on tenocyte adhesion and proliferation. To our knowledge, this study is the first to combine non-circular PCL fibers with collagen coating for use in tendon and ligament tissue engineering. With respect to the numerous requirements posed on the respective scaffolds, we aim to contribute to this research area by presenting a holistic approach of biologically improved melt-spun PCL scaffolds.

## 2. Results

### 2.1. Fiber Fabrication and Characterization

Fineness and mechanical properties of the round and snowflake-shaped PCL monofilaments are displayed in Table 1. Round monofilaments exhibit a slightly higher uniformity in terms of fineness as well as mechanical properties compared to the snowflake morphology.

**Figure 1:**
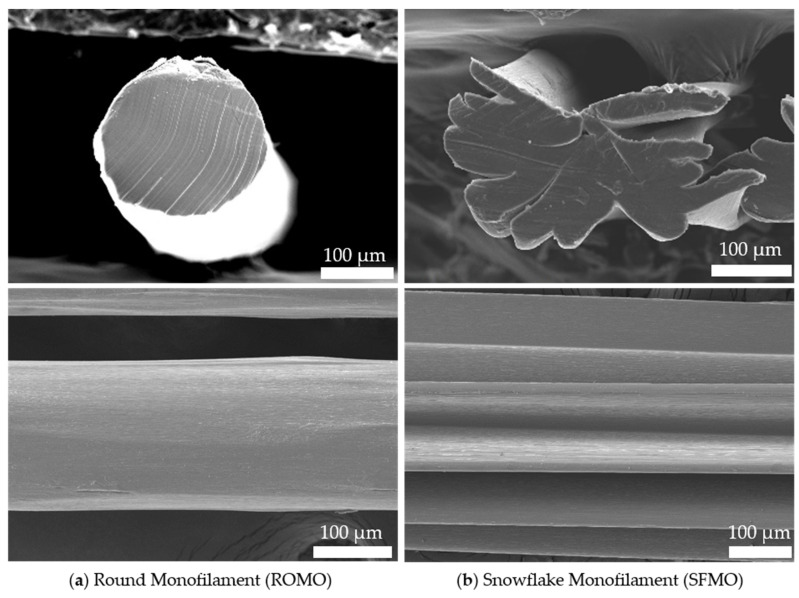
SEM images of (**a**) Round Monofilament (ROMO) and (**b**) Snowflake Monofilament (SFMO) [27].

The differing fiber topographies of ROMO and SFMO samples are depicted in Figure 1, with SFMO exhibiting axial grooves.

### 2.2. Morphological Characterization of the Braided Scaffolds

The braiding angle determines the braiding density, porosity, and pore sizes of the scaffolds. In this study, the scaffolds are braided with three different braiding angles.

**Figure 2:**
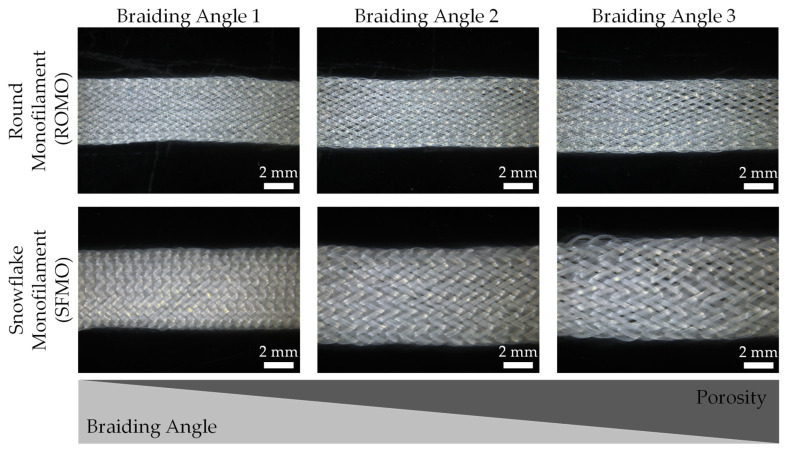
Microscopy images of the braided scaffolds made from monofilaments with round and snowflake cross-section. The porosity of the scaffolds decreases with increasing braiding angle.

In Figure 2, the braiding angles of all scaffolds are shown. Scaffolds braided from ROMO filaments exhibit a significantly higher value for braiding angle 1 (24.0° ± 2.3°) than for braiding angle 3 (16.5° ± 3.1°). Braiding angle 2 (22.0° ± 2.8°) differs also significantly from braiding angle 3. For SFMO scaffolds, braiding angle 1 (32.8° ± 2.2°) varies significantly from braiding angle 2 (26.5° ± 2.6°) and 3 (24.1° ± 3.3°). The angles 2 and 3 do not show any significant difference. The differences in the braiding angle with the same process parameters result from the larger circumference of the SFMO fibers and the surface topography.

Braiding density is defined as the number of interlacing points per length. The braiding densities of the different angles are compared within the same filament type.

**Figure 3:**
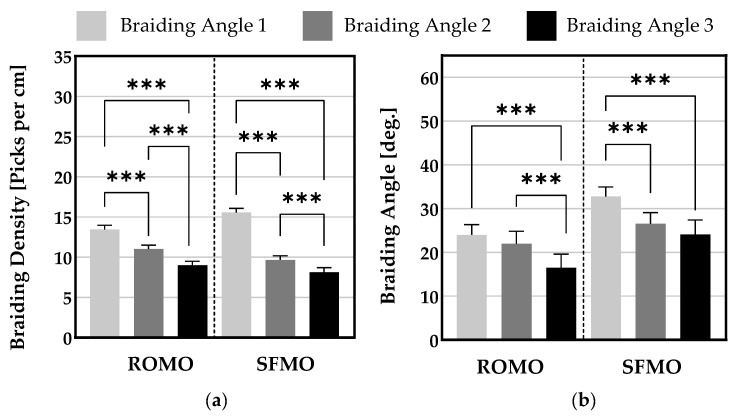
(**a**) Measured braiding density and (**b**) braiding angle of the scaffolds. (*** *p* < 0.001).

Figure 3 depicts the braiding densities of all scaffolds. All braiding densities within every filament type show a significant difference. The greatest values of braiding densities are given for braiding angle 1 and they linearly decrease in braiding angles 2 and 3, respectively.

The porosity of the braids made from round monofilaments ranges from 39.62% ± 4.38% in ROMO 1 to 44.62% ± 0.15% in ROMO 3. For scaffolds based on snowflake-shaped monofilaments, the porosity ranges from 40.57% ± 1.66% (SFMO 1) to 43.52% ± 1.43% (SFMO 2). The increase in porosity from a high braiding angle to a low braiding angle is more clearly observed in round fiber morphologies.

**Figure 4:**
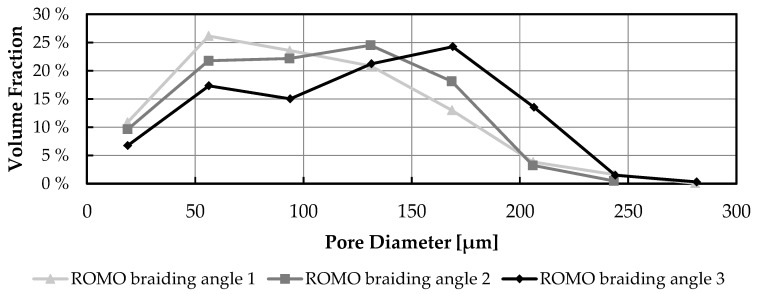
Volume fraction histogram of the scaffolds made from round filaments in the three different braiding angles.

The exemplary pore distribution of three ROMO scaffolds is shown in Figure 4. By changing the braiding angle, the peak of the pore distribution is shifted. With a higher braiding angle, the pore distribution is shown with a maximum at 56 µm. This shifts for braid angle 3 to 168 µm. For the SFMO scaffolds, a similar shift can be recorded.

The braided scaffolds consisting of 48 ROMO fibers show primary stabilities (maximum tensile load) between 323 ± 8 N at braiding angle 3 and 342 ± 10 N at braiding angle 1. The SFMO scaffolds vary between 447 ± 24 N at braiding angle 3 and 465 ± 25 N at braiding angle 2.

### 2.3. Biological Characterization

**Figure 5:**
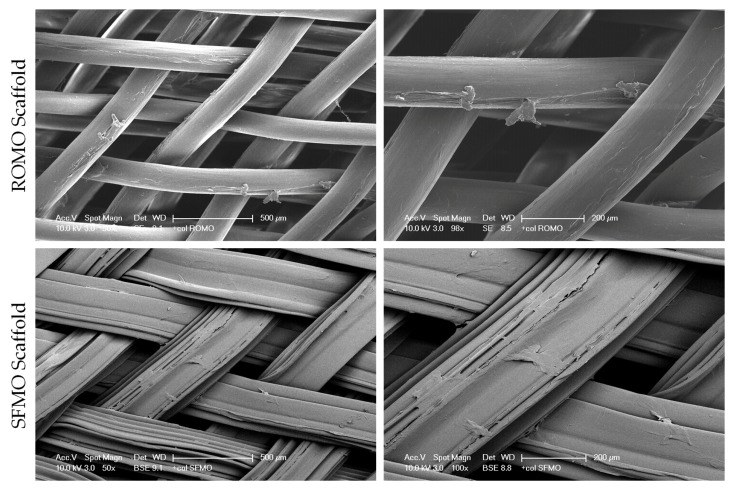
SEM images of the braided scaffolds from round monofilaments (ROMO) and snowflake monofilaments (SFMO), respectively, after seeding and in different magnifications.

The objective was to compare the PCL materials with various braiding angles, filament types, and collagen coatings by evaluating cell proliferation and viability, as well as the expression of tenogenic markers. The scaffold morphology after seeding is depicted in Figure 5.

#### 2.3.1. Cell Viability

**Figure 6:**
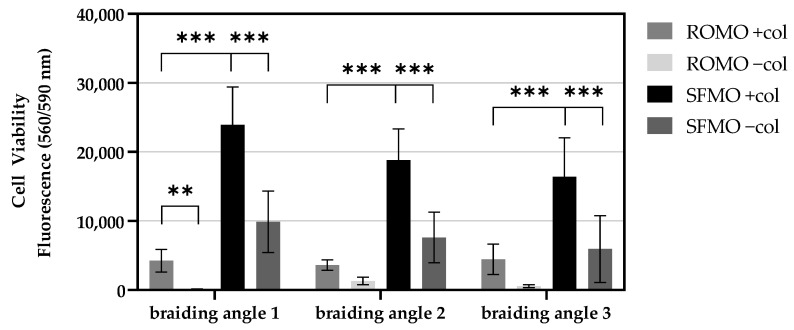
Cell viability at day 7 for collagen-coated and -uncoated scaffolds for all three braiding angles made from monofilaments with a snowflake (SFMO) and round (ROMO) cross-section. (** *p* < 0.01; *** *p* < 0.001).

The cell viability is correlated to the metabolic activity of cells. The scaffolds with collagen coating show significantly higher cell viability. In Figure 6, the fluorescence signal is depicted for ROMO and SFMO scaffolds after seven days of incubation time. For all braiding angles, the viability is significantly increased with collagen coating on the SFMO scaffolds. On average, the viability was increased by a factor of 2.55 by the collagen coating on SFMO scaffolds. ROMO scaffolds with and without show only a significant difference in viability for braiding angle 1.

By analyzing the effect of the filament type, an increase in cell viability by a factor 4.81 can be measured on SFMO scaffolds with collagen compared to ROMO scaffolds with collagen coating. SFMO shows significantly higher cell viability for all braiding angles by comparing the coated and uncoated scaffolds with the same braiding angle, respectively.

The scaffolds differ in their braiding angle and consequentially in their porosity. The cell viability shows a decrease on day 7 for SFMO scaffolds with and without collagen coating with an increasing braiding angle. In contrast, for ROMO scaffolds with a collagen coating, no significant difference was measured on day 7.

**Figure 7:**
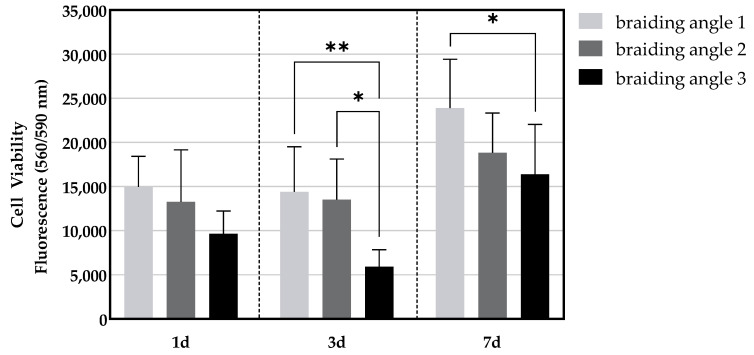
Cell viability at day 1,3, and 7 for collagen-coated scaffolds made from monofilaments with a snowflake (SFMO) cross-section for all three braiding angles. (* *p* < 0.05; ** *p* < 0.01).

The time course (day 1, 3, 7) of cell viability on the SFMO + col scaffolds is shown in Figure 7. On the first day of the incubation period, no difference in cell viability was observed between the three braiding angles in SFMO + col scaffolds. On the third day of the experiment, the viability of the cells in the scaffolds with braiding angles 1 and 2 was significantly higher than that of the cells in the scaffolds with braiding angle 3. On day 7, a significantly higher cell viability was measured for the lowest angle (braiding angle 1) compared to the highest braiding angle (braiding angle 3).

#### 2.3.2. Cell Proliferation

**Figure 8:**
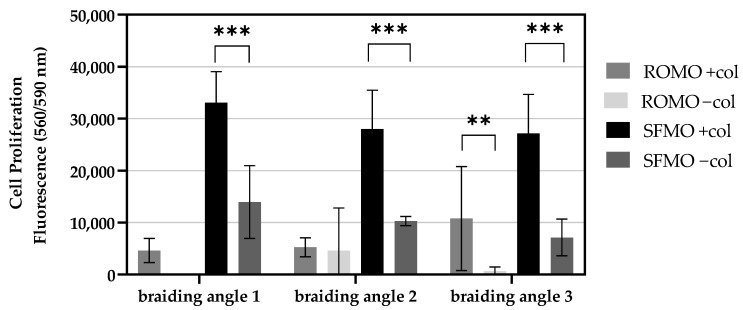
Fluorescence signal of the cell proliferation assay at day 7 for collagen-coated and -uncoated scaffolds for all three braiding angles made from monofilaments with a snowflake (SFMO) and round (ROMO) cross-section. (** *p* < 0.01; *** *p* < 0.001).

**Figure 9:**
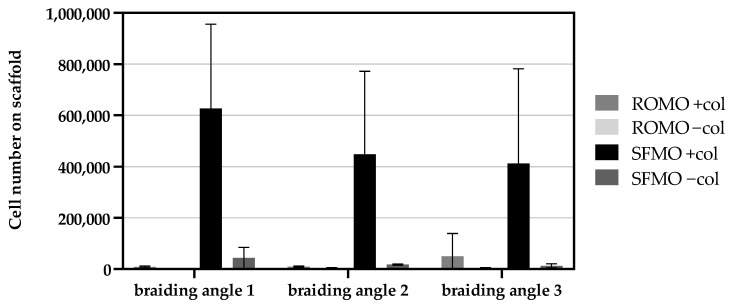
Cell number based on the cell proliferation assay at day 7 for collagen-coated and -uncoated scaffolds for all three braiding angles made from monofilaments with a snowflake (SFMO) and round (ROMO) cross-section.

The number of cells on the scaffolds is analyzed within all braiding angles (1–3) of the round and snowflake monofilament scaffolds with and without collagen coating (+/−col). Upon comparing cell numbers of different braiding angles within collagen-coated scaffolds, there were no significant differences exhibited (Figure 8). The cell numbers of SFMO scaffolds are the highest of all scaffolds, irrespective of braiding angles. In uncoated scaffolds, there are significant differences in cell numbers for SFMO scaffolds (Figure 9). The cell number of scaffolds with braiding angle 1 differs significantly from cell numbers in scaffolds with braiding angle 2 (*p*-value = 0.0016) and 3 (*p*-value = 0.0002). The data indicate that scaffolds braided from ROMO filaments demonstrate no statistically significant differences in values between the various braiding angles. The collagen coating has been demonstrated to enhance cellular attachment and proliferation in the SFMO scaffolds for all braiding angles compared to the uncoated scaffolds. For the ROMO scaffolds, the reduction in cell number is observed in col- groups, the difference being significant only at braid angle 3. By analyzing the cell proliferation regarding the fiber cross-section, the cell number of SFMO braided scaffolds reveals significantly higher values than ROMO braided scaffolds for every braiding angle. In scaffolds with braiding angle 1, SFMO scaffolds have the highest average cell number and differ significantly from the cell number of ROMO scaffolds (*p*-value = 0.0003). A significant difference between collagen-coated round and snowflake filaments can also be detected for braiding angle 2 (*p*-value = 0.0139) and for braiding angle 3 scaffolds (*p*-value = 0.0494). In uncoated scaffolds, the monofilaments appear to be significantly different for braiding angle 1 (Figure 9), but not for braiding angles 2 and 3, although there is a slight tendency for SFMO scaffolds to hold higher cell numbers.

#### 2.3.3. Immunofluorescence

**Figure 10:**
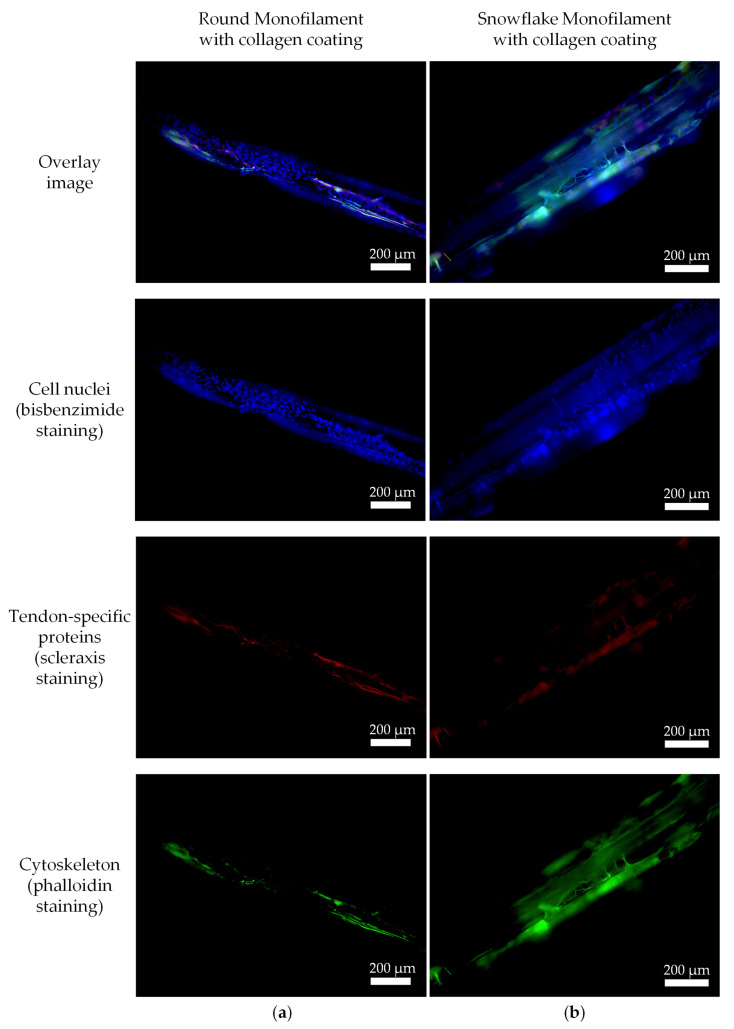
Fluorescence-stained (bisbenzimide, phalloidin, and scleraxis) cells on single filaments of (**a**) round and (**b**) snowflake cross-sections.

Immunofluorescence staining was performed to verify the cell attachment and expression of tenogenic markers of the cells. The scaffolds were stained with an antibody against tenomodulin a and scleraxis. Phalloidin dye was used to visualize the cytoskeleton and cell elongation, as well as bisbenzimide for nuclear staining. In Figure 10a, a single round monofilament seeded with tenocytes is illustrated. The overlaid picture shows the cells across the surface of the filament. Scleraxis can be detected within the cells. The nuclei staining reveals many dyed spots (nuclei) on the surface of the filament. Furthermore, the cytoskeleton is longitudinally stretched over the surface.

A single snowflake monofilament is depicted in Figure 10b. The snowflake topology is indicated by the micro-grooved structure of the surface. The overlay picture shows several cells that align in fiber direction along the grooves and form cell protrusions between the grooves. The red channel indicates the presence of scleraxis in these cells.

Collagen-coated and -uncoated scaffolds with a braiding angle 2 of each filament type were seeded with tenocytes and incubated for seven days. The samples were treated with antibodies for tenomodulin, the cytoskeleton was stained with phalloidin, and the nuclei with bisbenzimide. The shown pictures are overlay pictures with all three channels combined.

**Figure 11:**
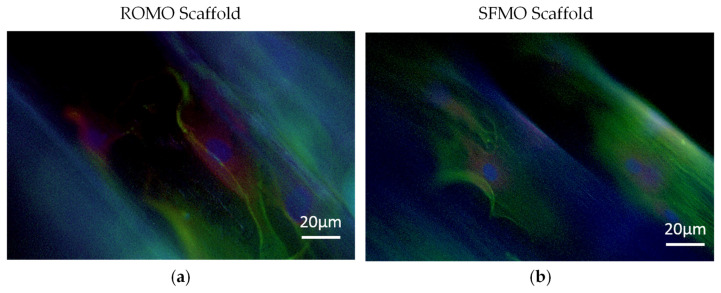
Fluorescence-stained (bisbenzimide, phalloidin and tenomodulin) cells on braided scaffold out of (**a**) round monofilaments and (**b**) snowflake monofilaments.

Figure 11 shows the immunofluorescent pictures of collagen-coated scaffolds made from ROMO and SFMO filaments, respectively. The autofluorescence of PCL shows the scaffold structure in blue. The collagen-coated filaments reveal attached and grown cells on the surface. The nuclei as well as the cytoskeleton and the perinuclear protein tenomodulin are clearly visible (Figure 11b). The cells expand along the axis of the filaments, showing an elongated cell body and cell protrusions spreading between the grooves of the surface topology in case of SFMO. On uncoated scaffolds, a lower cell number was detected, but the cells also express an elongated cytoskeleton.

## 3. Discussion

Scaffolds for tendons or ligaments should meet numerous requirements including mechanical, degradational, morphological, process-related, and biological requirements [31,32,33]. As shown in vitro, in the equine joint defect model and in clinical studies, the incorporation of autologous cells into polymer-based scaffolds allows the formation of mesenchymal repair tissue [34,35,36]. In addition, resorbable polymer-based scaffolds are initially stable and provide easy handling during surgery and secure and mechanically stable fixation of grafts or implants, e.g., by pinning, suturing or anchoring in the bone. Therefore, resorbable polymer-based scaffolds may also be promising biomaterials for cell-based tendon repair approaches [37]. Using melt-spun PCL filaments braided into a macroporous textile scaffold is a promising approach to fulfill these requirements [27,28,29]. This technique can be used to fabricate human-scaled scaffolds with appropriate primary stability, stiffness, and degradation kinetics [27]. This is also applicable to the fibers presented in this study, since their mechanical properties are comparable to those of the fibers used in the aforementioned studies. However, the cell response is crucial for the functionality of the scaffold. The topography of the filament has a critical role in determining its porosity, density, and biomechanical properties; however, the effect of filament topography on cellular response has been poorly investigated [38]. The attachment of tenocytes to filaments in relation to filament topography were investigated. Furthermore, we investigated if the collagen coating optimizes the cell adherence and proliferation on different types of filaments.

Non-circular fibers provide a greater surface area for cell attachment and axial grooves as potential growth guidance. The snowflake shape was selected in this study as an example for highly serrated fiber cross-sections. Park et al. studied cell proliferation on extruded PCL strands with circular, triangular, and cruciform cross-sections, respectively. Compared to the circular reference, triangular and cruciform samples showed 50% and 112% increased cell proliferation (MG-63 osteosarcoma cells) [26]. Only marginal improvement of murine TGF-β-type-2 receptor (Tgfbr2)-expressing progenitor cell proliferation was observed by Ramakrishna et al. who used PLA multifilaments with axial grooves in scaffolds for the bone–tendon junction. However, an increasing alignment as well as attachment of the cells within the axial grooves were noticed [24]. Similarly, Lu et al. discovered that over 85% of the cell nuclei were aligned in parallel with the axis of their PLA and PET fiber exhibiting capillary channels [25]. As is in the present study, the effect of different fiber cross-sections (profiles) with/without collagen coating on the cell response was investigated by Tai et al. PET fibers (with and without additional calcium phosphate incorporation) were melt-spun in four different profiles (circular, I-, Y-shaped, cruciform) and consequently woven, collagen coated, dried, and rolled into an artificial ligament. The Y-shaped fibers showed significantly improved proliferation of rat bone marrow mesenchymal stem cells (MSC) compared to their circular and I-shaped counterparts, especially in combination with the collagen coating. A lesser improvement of cell proliferation was observed with the cruciform fibers, while the I-shaped profile has no significant effect compared to the circular fibers. The collagen coating improved the cell response for all fiber profiles and all time points (3 d, 7 d, 14 d) [39]. In our previous studies, we investigated the cell response of human MSCs on PCL snowflake multifilaments and circular monofilaments with and without an additional surface modification using Chitosan-graft-PCL. In the course of this, a clear tendency towards a cell orientation parallel to the fiber axis as well as the formation of cell protrusions within and across the grooves of the snowflake multifilaments were observed [27,29].

The snowflake shape was part of another recent study in which PCL braided scaffolds from snowflake-shaped and circular monofilaments, respectively, were investigated regarding the response of human MSCs. Therein, the snowflake exhibited the highest cell viability, however, only in combination with increased scaffold density mediated through the higher of two braiding angles [30]. In line with this observation, the braided scaffolds with higher braiding angles in the present study also exhibited an improved cell response for the coated and uncoated snowflake monofilaments, while no such effect was observed for the circular counterparts. Summarizing the main findings of topographical performance, cell alignment with the fiber axis and improved cell attachment are often observed, while the effect on cell proliferation varied widely between studies, suggesting the interplay of many factors, i.e., coating. The results of the present study are consistent with the literature as well as our recent works in terms of a tendency to cell orientation parallel to the fiber axis as well as improved cell attachment in the snowflake group. Interestingly, our data show that the snowflake cross-section has a markedly improved effect on cell attachment and proliferation of tenocytes compared to the circular cross-section.

The hydrophobic surface of the synthetic materials hindered cell attachment, so to enhance the bioactive surface of the scaffolds, they were coated with collagen.

Collagen is the predominant component of the ECM in tendons and ligaments and is expressed by the cells themselves, although only 5% of the tissue is filled with cells. Type I collagen, in particular, has a strong positive effect on cell differentiation and proliferation.

Various studies have shown the beneficial effect of integrating the main component of the extracellular matrix, like collagen in the scaffold. Gögele et al., for instance, functionalized embroidered scaffolds from P(LA-CL)- and PLA filaments with cross-linked collagen foam and gas-phase fluorination. The functionalized samples showed improved cell colonization and penetration towards the inner parts of the three-dimensional scaffold [16]. Sharifi-Aghdam et al., who combined knitted silk scaffolds with blended collagen and PU nanofibers, also observed the beneficial effects of using collagen in the form of improved cellular attachment as well as proliferation [18]. Ide et al. also stated positive results by integrating collagen in 3D-braided PLLA scaffolds for anterior cruciate ligament (ACL) reconstruction. The infiltration with collagen resulted in an improved cell seeding with human ACL-derived cells as well as promoted cell immigration and neo-angiogenesis in the rat model [17]. Similarly to the study conducted by Tai et al., a combination of topographical stimuli by using a non-circular fiber cross-section and collagen coating appeared to lead to the best cell response [39]. In line with the positive observations of using collagen, the collagen coating in the present study resulted in a substantial improvement of cell viability and proliferation for all braiding angles and both fiber morphologies. The combination of PCL as a biomechanically adaptive scaffold with collagen coating as the main component of the ECM leads to an improvement in the cell-seeded scaffold.

## 4. Materials and Methods

### 4.1. Fiber Fabrication

**Figure 12:**
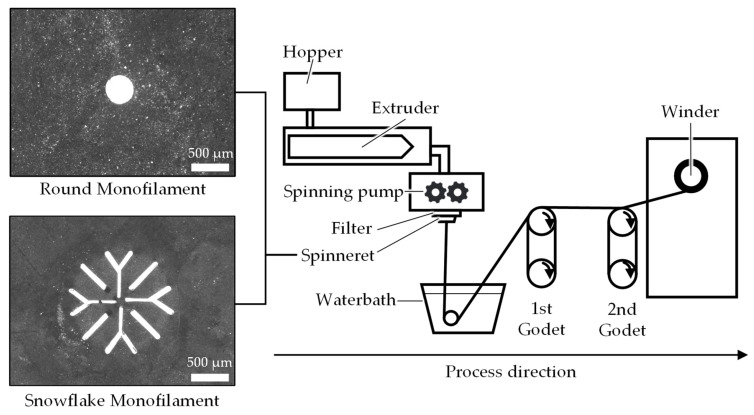
Schematic of the melt spinning process using two types of spinnerets [27].

Poly-ε-caprolactone (Capa^®^ 6800, Ingevity Corp., North Charleston, SC, USA) was melt-spun using a single-screw extruder spinning plant (Fourné Polymertechnik GmbH, Alfter, Germany) in a setup consistent with previous studies [27,28,29]. Round and snowflake-shaped monofilaments (abbreviated as ROMO and SFMO) were fabricated using respective spinnerets (see Figure 12). Extrusion temperatures were 222 °C for ROMO and 235 °C for SFMO in order to compensate differences in shear stresses between the spinnerets. A draw ratio (DR) of 6.83 was applied using two godet pairs before winding (SAHM 260XE, Georg Sahm GmbH & Co. KG, Eschwege, Germany).

### 4.2. Fiber Characterization

Fineness was measured in accordance with DIN EN 13392 [40] (*n* = 3). Mechanical properties (*n* = 30) were determined in uniaxial tensile tests based on DIN EN 13895 using a tensile testing machine (STATIMAT 4U, Textechno Herbert Stein GmbH & Co. KG, Mönchengladbach, Germany). The samples were subjected to standard conditions for textile testing following DIN EN ISO 139 (20 ± 2 °C and RH 65 ± 4%) for at least 24 h. The filament topography was investigated via scanning electron microscopy (FlexSEM 1000 II, Hitachi Ltd., Tokyo, Japan) after coating the samples with a thin gold layer using a sputter coater (Cressington Sputtercoater 108, Tescan GmbH, Dortmund, Germany).

### 4.3. Scaffold Production

Scaffolds were produced with a circular 48 carrier braiding machine (HS 80/48, Körting Nachf. Wilhelm Steeger GmbH & Co. KG, Wuppertal, Germany) equipped with fine wire bobbins with three deflection rollers. All scaffolds consist of 48 filaments each. The braiding angle is adjusted by changing the distance of the braiding point to the bobbins. The following values were set during the braiding process: Braid angle 1: *L* = 11.5 cm; Braid angle 2: *L* = 18 cm; Braid angle 3: *L* = 27.5 cm. Braiding angle 1 is the widest angle, followed by braiding angle 2 as the intermediate angle and braiding angle 3 as the smallest. A rotational speed of 30 rpm was used.

### 4.4. Scaffold Morphology

The braiding angles of the scaffolds were investigated using light microscopy pictures. The angles were measured using the software CorelDRAW (Version 2022, Corel Corporation, Ottawa, ON, Canada). Images of three different sites of each scaffold were taken. Per image, five angles were measured. The mean of 15 measured angles per scaffold type is given with standard deviation.

The braiding density complies with the interlacing points per length and was evaluated on three different sites of the scaffolds. The results were calculated as the mean of three values per scaffold including standard deviation. The braiding density is given in picks per cm.

**Figure 13:**
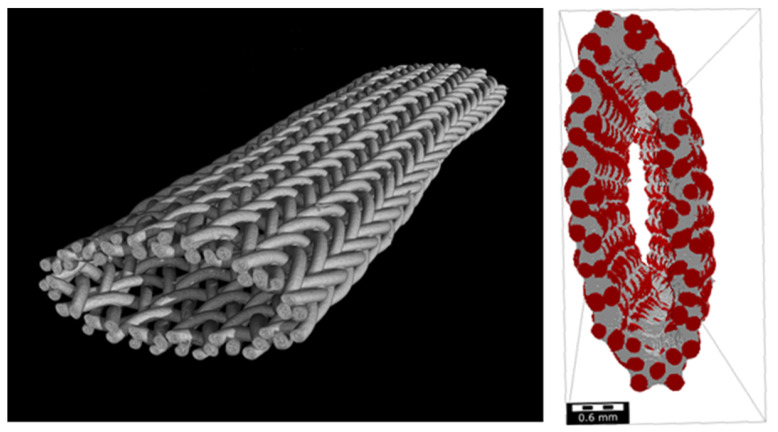
Example of a µ-CT scan (from a braided scaffold consisting of round monofilaments) including the area fraction of pores (gray) and fibers (red).

Porosity and pore size distribution of the scaffolds were analyzed using µ-CT scans and the software GeoDict (Version 2023, Math2Market GmbH, Kaiserslautern, Germany). Three scans of each scaffold type were investigated. Figure 13 shows an exemplary scan. The porosity was determined based on a 3D model of the µ-CT scans. For this purpose, the fibers are masked as solid and the spaces between them as permeable. The cavity inside is not considered. Only the pores between the fibers, which are exposed to cell contact, are analyzed. The spaces are then filled so that the porosity can be calculated from the difference to the fibers.

### 4.5. Isolation and Culturing of Tenocytes

Rat tenocytes were isolated from Achilles tendons of wild-type Wistar rats (200–500 g). The excised tendons were washed in phosphate-buffered saline (PBS). The tendon was transferred in 2 milliliter (mL) pure Dulbecco’s Modified Eagle Medium (DMEM) high glucose (GIBCO^®^, Invitrogen, Waltham, MA, USA). The tendon was split across the fiber direction into small pieces after careful removal of the epitendineum. A total of 200 microliters (μL) of Trypsin/EDTA (10×) was added (1:10) after an incubation time of five minutes at 37 °C. Subsequently, the tendon pieces were washed and cultivated in DMEM supplemented with 50% fetal calf serum (FCS), 100 units/mL penicillin-streptomycin and 100 μg/mL fungizone (PSF) and incubated at 37 °C in a Petri dish. After approximately five days, tenocytes continuously migrated from this explant and adhered to Petri dishes. The FCS will be subject to a continuous reduction, reaching a final value of 10%. After 70–80% confluence, cells were removed using 1% trypsin (5×; Gibco, Invitrogen) and cultivated in culture medium containing 10% FCS and PSF. The cells were used from the second through fourth passages.

### 4.6. Collagen Coating

The scaffolds were cut with micro-serrated scissors to 9 mm length. The braided scaffolds and filaments were sterilized for 15 min in 70% (*v*/*v*) Ethanol (EtOH) and washed twice with PBS afterwards. For collagenization, the scaffolds were dipped in a solution with sterile 10% (*v*/*v*) Collagen-A (col) (1:9 in PBS) and incubated for four hours at room temperature under sterile conditions.

### 4.7. Cell Viability Assay

The CellTiter-Blue (CTB) cell viability assay is based on the principle of reduction reactions. The assay was performed with collagen-coated (+col) and uncoated (−col) braided PCL material with different braiding angles. The assay contained three biological replicates (three cellularized scaffolds per filament type and +/−col) and two technical replicates (duplicates of the measured reagent). The assay was performed twice for every braiding angle. Hence, six biological replicates are considered in the evaluation. Based on preliminary experiments, the scaffolds were cellularized with 35,000 cells per scaffold. The assay was performed after 24 h, 72 h, and seven days of incubation. To avoid any positive reaction from adherent cells on the well surface, the scaffolds were transferred directly into the new 24-well plate just prior to measurement. The CTB reagent was combined with the culture medium (free of phenol red) at a ratio of 1:5. A solution of 350 μL of diluted CTB was then administered to each scaffold. After incubation of 120 min, 70 μL of the solution was pipetted twice into a 96-well plate. Fluorescence was quantified at 560 nm excitation and 590 nm emission using a fluorescence microplate reader (Infinite M200, TECAN, Männedorf, Switzerland). Diluted CTB served as a blank sample to detect the autofluorescence of the CellTiter Blue reagent. The emitted fluorescence signal is proportional to the number of viable cells on the scaffold.

### 4.8. Cell Proliferation

The CyQUANT assay is based on the emitted fluorescence of a green fluorescence dye (CyQUANT GR dye) when bound to cellular nucleic acids. The fluorescence signal is proportional to the number of nucleic acids and, therefore, to the number of cells on the scaffolds. The assay was performed with collagen-coated (+col) and -uncoated (−col) braided PCL material with different braiding angles. The assay contained three biological replicates (three cellularized scaffolds per filament type and +/−col) and two technical replicates (duplicates of the measured reagent). The assay was performed twice for every group. Hence, six biological replicates are considered in the evaluation. The scaffolds used for the seven-day CTB assay were washed three times with PBS to remove the CTB reagent. In accordance with the manufacturer’s instructions, cell lysis buffer (component B) was combined with ultra-pure water (DEPEC) in a 1:20 ratio. A total of 200 μL cell lysis buffer was added to each scaffold. The scaffolds were stored at −80 °C overnight. The CyQUANT GR dye was added to the diluted lysis buffer (1:200 with cell lysis buffer) and 200 μL of the resulting mixture was applied to each scaffold. A total of 70 μL of the supernatant from each scaffold were transferred into a 96-well plate in duplicate and measured with excitation at 480 nm and emission detection at 540 nm using a fluorescence microplate reader (Infinite M200, TECAN, Männedorf, Switzerland). The pure CyQUANT mixture served as a blank sample to detect the autofluorescence of the dye. The emitted fluorescence signal is proportional to the total number of cells on the scaffold. The cell numbers were calculated with a linear regression of the standard curve.

### 4.9. Immunofluorescence Staining

For cellularization, 50,000 cells were added to each braided PCL in 24-well plates. The medium was replaced every two to three days. After seven days of incubation, the samples were washed twice with PBS. The samples were fixed in a 3.7% formalin solution for a duration of 20 min at room temperature, after which they were washed five times in order to remove the formalin from the sample. For permeabilization, the samples were washed three times for five minutes with 0.1% Triton X-100 + hydroxymethyl aminoethane (TRIS) buffer. After washing, the samples were incubated for 30 min at room temperature in a blocking solution (1% Bovine serum albumin (BSA) in TRIS buffer). The primary antibody was diluted (tenomodulin 1:250 (Sc49325), scleraxis 1:150 (Sc-87425) in TRIS + 1% BSA and added to the sample, which was then incubated overnight at 4 °C in a humidity chamber. The next day, the samples were washed three times for ten minutes in TRIS buffer. Next, the first secondary antibody (AlexaFluor 555, anti-goat (against tenomodulin); AlexaFlour 594, anti-mouse (against Scleraxis) was diluted 1:400 in TRIS + 1% BSA and combined with a solution of Alexa Fluor™ (Thermo Fisher Scientific, Waltham, MA, USA) 488 Phalloidin (A12379) (1:50) were of 1:50 added to each sample. After an incubation time of 1 h, the samples were washed three times for 10 min with TRIS buffer. The nucleus was stained with bisbenzimide (1:10,000 in PBS) for 10 min. The samples were washed four times with aqua dest. The observation of the samples was conducted using a fluorescence microscope Keyence BZ-9000 (Keyence Corp., Osaka, Japan).

### 4.10. Statistical Evaluation

The statistical evaluation was carried out using GraphPad Prism 8. The *p*-value was set to 0.05 as the significance level. The significance was determined using a one-sided ANOVA test for statistics covering one variable and a two-sided ANOVA test for statistics with two variables. The graphs are presented with the mean and standard deviation indicated. The significance levels are given as follows: non-significant for *p*-values > 0.05; * for *p*-values < 0.05; ** for *p*-values < 0.01; and *** for *p*-values < 0.001. *p*-values are given when the highest significance level (***) is not reached in the statistical evaluation.

## 5. Conclusions

Tissue engineering of tendons and ligaments offers a promising way to overcome the shortcomings of current therapies but imposes multiple requirements on the scaffold used. We compared the viability of seeded tenocytes on the PCL fibers designed in round and snowflake cross-sections and with different braiding angles. Cell attachment on snowflake fibers was significantly higher than on round fibers.

Optimization of tenocyte seeding was obtained by coating the scaffold with collagen. Using non-circular fiber cross-sections as well as applying a collagen coating resulted in pronounced improvements in cell viability and proliferation, especially when combined. Interestingly, we were able to show that a higher scaffold density appeared to lead to a superior cell response.

The snowflake morphology exhibited an interrelation with the scaffold density so that its effect was more pronounced on dense scaffolds fabricated with a high braiding angle. Cellular orientation tended to align with the fibers’ orientation. This study indicates the immense potential for improvement of the cell response of mechanically competent scaffolds through these modifications. It also shows the complexity of the interplay of different factors in the cell response, which should be addressed further systematic investigations.

## Figures and Tables

**Table 1:** Fineness and mechanical properties of PCL monofilaments.

Fiber	Fineness	Tensile Strength		Max. Load	Elongation at Max. Load
	[dtex]	[cN/tex]	[MPa] *	[N]	[%]
Round Monofilament (ROMO)	378 ± 0.7	40.5 ± 1.9	462	15.3 ± 0.7	68.1 ± 3.8
Snowflake Monofilament (SFMO)	388 ± 5.5	36.8 ± 5.1	421	14.3 ± 2.0	59.7 ± 9.0

* Calculated assuming a constant density of ρ_PCL_ = 1.145 g/cm^3^.

## Data Availability

Data are contained within the article.
